# Absorption and thermodynamic properties of CO_2_ by amido-containing anion-functionalized ionic liquids†

**DOI:** 10.1039/c8ra07832g

**Published:** 2019-01-14

**Authors:** Yanjie Huang, Guokai Cui, Huiyong Wang, Zhiyong Li, Jianji Wang

**Affiliations:** Henan Key Laboratory of Green Chemistry, Collaborative Innovation Center of Henan Province for Green Manufacturing of Fine Chemicals, Key Laboratory of Green Chemical Media and Reactions, Ministry of Education, School of Chemistry and Chemical Engineering, Henan Normal University Xinxiang Henan 453007 China jwang@htu.cn

## Abstract

In this contribution, two kinds of amido-containing anion-functionalized ionic liquids (ILs) were designed and synthesized, where the anions of these ILs were selected from deprotonated succinimide (H-Suc) and *o*-phthalimide (Ph-Suc). Then, these functionalized ILs were used to capture CO_2_. Towards to this end, solubility of CO_2_ in the ILs was determined at different temperatures and different CO_2_ partial pressures. Based on these data, chemical equilibrium constants of CO_2_ with the ILs were derived at different temperatures from the “deactivated IL” model. The other thermodynamic properties such as reaction Gibbs energy, reaction enthalpy, and reaction entropy in the absorption were also calculated from the corresponding equilibrium constant data at different temperatures. It was shown that these anion-functionalized ILs exhibited high CO_2_ solubility (up to 0.95 mol CO_2_ mol^−1^ IL) and low energy desorption, and enthalpy change was the main driving force for CO_2_ capture by using such ILs as absorbents. In addition, the interactions of CO_2_ with the ILs were also investigated by ^1^H NMR, ^13^C NMR, and FT-IR spectroscopy.

## Abstract 2

Amido-containing anion-functionalized ionic liquids exhibit high CO_2_ absorption capacity and low desorption energy.

## Graphical abstract

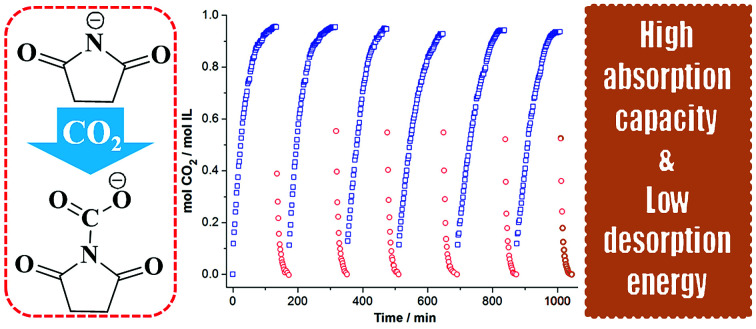

## 1. Introduction

In recent decades, the excessive accumulation of carbon dioxide (CO_2_) in the atmosphere has attracted widespread concern due to the greenhouse effect.^1^ In order to solve this problem, carbon capture and storage (CCS) through sustainable and green methods has become a hot topic in recent decades.^2^ Although aqueous alkanolamine solutions have been used in industry for nearly a century as a kind of chemical absorbent for CO_2_ in flue gas from power plants through the formation of ammonium carbamate, the main disadvantage of this process is the high energy consumption for the regeneration and recycling of the absorbent, which accounts for 30% of the energy of the power plant.^3,4^ Thus, alternative CCS methods for highly efficient and reversible capture of CO_2_ are desired.

In recent years, considerable attention has been focused on using ionic liquids (ILs), especially functionalized ILs, as a kind of competitive alternative green absorbent for CO_2_ capture. These liquid materials provide some unique advantages such as reduced volatilization and improved regeneration.^5–9^ In light of the reaction mechanism of CO_2_ in alkanolamine solutions and the outstanding properties of ILs, Davis *et al.*^10^ first reported imidazolium ILs with amino-grafted cation for the chemical absorption of CO_2_. Since the anion plays a key role in carbon capture, worldwide researchers have developed many kinds of task-specified ILs with the functionalized anions such as amino acids,^11–14^ azolates,^15–20^ phenolates,^7,21–23^ and acetate.^24^ Compared with conventional ILs, these anion-functionalized ILs typically have high CO_2_ absorption capacity and selectivity. It is highly encouraged that novel kinds of other anion-functionalized ILs should be developed and used in CCS process.

Thermodynamic properties of CO_2_ – functionalized IL systems are essential to guide the design of new functionalized ILs for CO_2_ capture. Up to now, great efforts have been devoted to the studies on the thermodynamic properties of CO_2_ chemical absorption by functionalized ILs. For instance, Brennecke *et al.*^25,26^ proposed “deactivated IL” model and “two-reaction” model to analyze the thermodynamic properties of CO_2_ in amine-functionalized ILs. Wang *et al.*^27^ reported that anion-functionalized [P_66614_][p-AA] and [P_66614_][p-ANA] had higher absorption capacity and lower reaction enthalpy because of entropic effects. Hu *et al.*^28^ systematically studied the thermodynamic properties of CO_2_ capture in low-viscous fluorine-substituted phenolic ILs through the “deactivated IL” model. Thus, it is very important to expand the thermodynamic studies of different types of functionalized ILs.

**Fig. 1:**
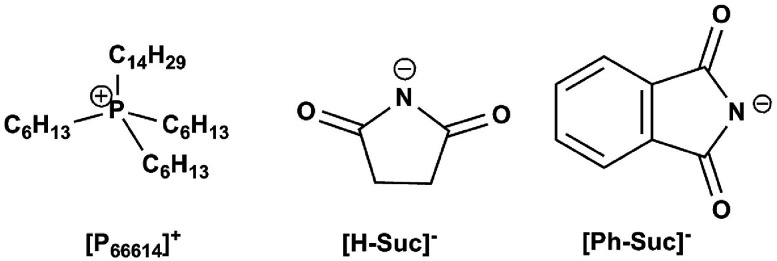
Chemical structures of ILs used in this work for CO_2_ capture.

In this work, we designed and synthesized two kinds of amido-containing anion-functionalized ILs (see Fig. 1) for CO_2_ capture. Solubility of CO_2_ in these functionalized ILs was determined at different temperatures and different CO_2_ partial pressures. From these data, the equilibrium constants for the reaction of CO_2_ with these ILs were calculated from a modified “deactivated IL” model as a function of temperature. Then, the Gibbs energy, enthalpy and entropy change were reported for the process of CO_2_ capture. It was shown that high CO_2_ absorption capacity and low energy consumption regeneration of the ILs could be achieved by these anion-functionalized ILs. From the viewpoint of chemical thermodynamic, enthalpy change is the main driving force for CO_2_ capture by using such ILs as absorbents.

## 2. Experimental section

### 2.1. Materials

CO_2_ and N_2_ were purchased from Beijing Oxygen Plant Specialty Gases Institute Co., Ltd. with a purity of 99.999% and 99.9993%, respectively. Trihexyl(tetradecyl)phosphonium bromide ([P_66614_][Br], 97%) and *o*-phthalimide (Ph-Suc, 98%) were obtained from J&K Scientific, while succinimide (H-Suc) was supplied by Sigma-Aldrich. An anion-exchange resin (Amersep 900 OH) was purchased from Alfa Aesar. All these substances were used as received.

### 2.2. Preparation of the ionic liquids

Anion-functionalized ILs were simply produced through the neutralization of different kinds of acylamides and an ethanol solution of trihexyl (trtradecyl)phosphonium hydroxide ([P_66614_][OH]) at room temperature according to the procedures described in literature,^29,30^ where [P_66614_][OH] was prepared from [P_66614_][Br] by anion-exchange method using ethanol as the solvent.^31^ In a typical synthesis of [P_66614_][H-Suc], equimolar H-Suc was added to the ethanol solution of [P_66614_][OH], and the mixture was then stirred at room temperature for 24 h. Then, ethanol and water were evaporated at 333 K under reduced pressure. The as-prepared [P_66614_][H-Suc] was dried with P_2_O_5_ under vacuum at 333 K for 24 h to remove possible residual moisture before use. The chemical structures of these ILs were confirmed by ^1^H NMR, ^13^C NMR and FT-IR spectra, and the data were listed in the ESI.†

### 2.3. Determination of CO_2_ solubility

Solubility data of CO_2_ in these ILs were measured by gravimetric method.^32^ In a typical measurement, CO_2_ was bubbled through about 1.0 g IL loaded in a glass container with an inside diameter of 12 mm, and the gas flow rate monitored by gas rotameter was about 60 ml min^−1^. The glass container was partly immersed in a water bath which was maintained at the given temperature with temperature uncertainty of ± 0.1 K. During gas absorption, weight of the sample was determined at regular intervals by an electronic balance with an accuracy of ± 0.1 mg until it became constant. At this stage, the adsorption equilibrium of CO_2_ in the IL was reached, and the solubility of CO_2_ in the IL could be calculated. For the absorption of CO_2_ under different partial pressure, CO_2_ was diluted by N_2_ gas and the given partial pressure was produced by controlling the flow rate ratio of CO_2_ and N_2_.

### 2.4. Characterization of the ionic liquids

^1^H NMR and ^13^C NMR spectra were determined on a Bruker spectrometer (400 MHz) in DMSO-d_6_ with tetramethylsilane (TMS) as the standard. FT-IR spectra were recorded using a Nicolet 470 FT-IR spectrometer. The structures of ([P_66614_][H-Suc] and [P_66614_][Ph-Suc]) before and after CO_2_ absorption were confirmed by NMR and FT-IR spectroscopy. The water contents in these ILs after drying, determined with Karl Fischer Titration (Mettler Toledo DL32, Switzerland), was lower than 0.1 wt%. The residual halide content, as determined by combining a Br^−^ selective electrode (Shanghai Precision & Scientific Instrument Co. Ltd.) with a saturated calomel electrode (Shanghai Precision & Scientific Instrument Co. Ltd.), was less than 0.0005 mol per kilogram.

## 3. Results and discussion

### 3.1. Solubilities of CO_2_ in the ionic liquids

**Fig. 2:**
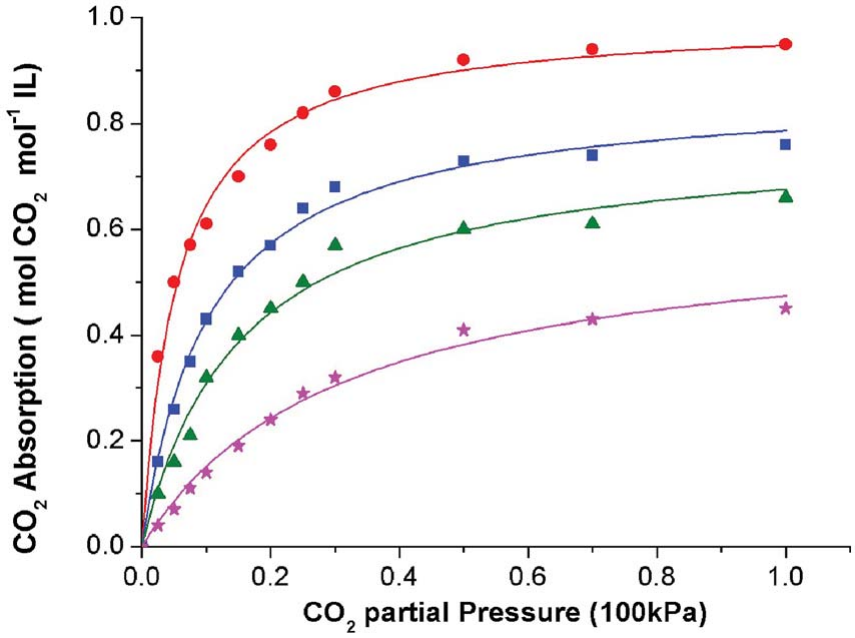
The absorption isotherms of the CO_2_-[P_66614_][H-Suc] system at different temperatures: 308.15 K, (●) 313.15 K, (■) 318.15 K, (▲) 323.15 K, (★) the curves were fittings from eqn (4).

**Fig. 3:**
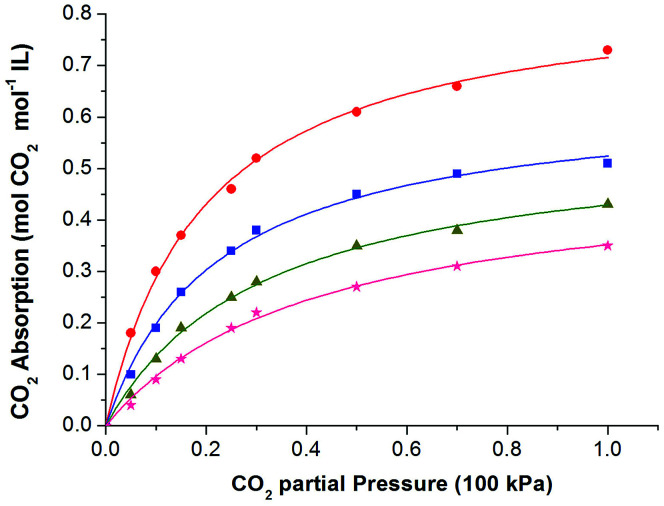
The absorption isotherms of the CO_2_-[P_66614_][Ph-Suc] system at different temperatures: 308.15 K, (●) 313.15 K, (■) 318.15 K, (▲) 323.15 K, (★) the curves were fittings from eqn (4).

At the beginning, solubility of CO_2_ in these ionic liquids was measured at 308.15 K under atmospheric pressure. The results showed that up to 0.95 mol CO_2_ per mol of IL could be achieved by these functionalized ILs, indicating good absorption performance of the absorbents. Thus, the solubility of CO_2_ in these ILs was determined at 308.15 K, 313.15 K, 318.15 K, and 323.15 K under different CO_2_ partial pressures. Table S1 and S2 (see ESI†) listed the solubility data of CO_2_ in [P_66614_][H-Suc] and [P_66614_][Ph-Suc] at various temperatures and CO_2_ partial pressures. For the sake of easy understanding, these data were shown in Fig. 2 and 3, respectively. It can be seen that the solubility of CO_2_ in the ILs increased with increasing partial pressure in the range of low pressure. For instance, the solubility of CO_2_ in [P_66614_][H-Suc] at 308.15 K was 0.61 mol CO_2_ mol^−1^ IL under 10 kPa of CO_2_ partial pressure, while it increased to 0.95 mol CO_2_ mol^−1^ IL under 100 kPa of CO_2_ partial pressure. However, Fig. 2 and 3 exhibited a nonlinear absorption trend with the increase of CO_2_ partial pressure. This indicates that the absorption of CO_2_ in these ILs was mainly carried out through chemical absorption. On the other hand, when the temperature increased from 308.15 K to 323.15 K, the molar ratio of CO_2_ to [P_66614_][H-Suc] reduced from 0.61 to 0.14 under 10 kPa of CO_2_ (Fig. 2). This result suggests that the captured CO_2_ could be facilely released by mild heating. Thus, the CO_2_ absorption process by these ILs was characterized by high CO_2_ absorption capacity and low energy desorption.

### 3.2. Chemical absorption mechanism of CO_2_ in the ionic liquids

**Fig. 4:**
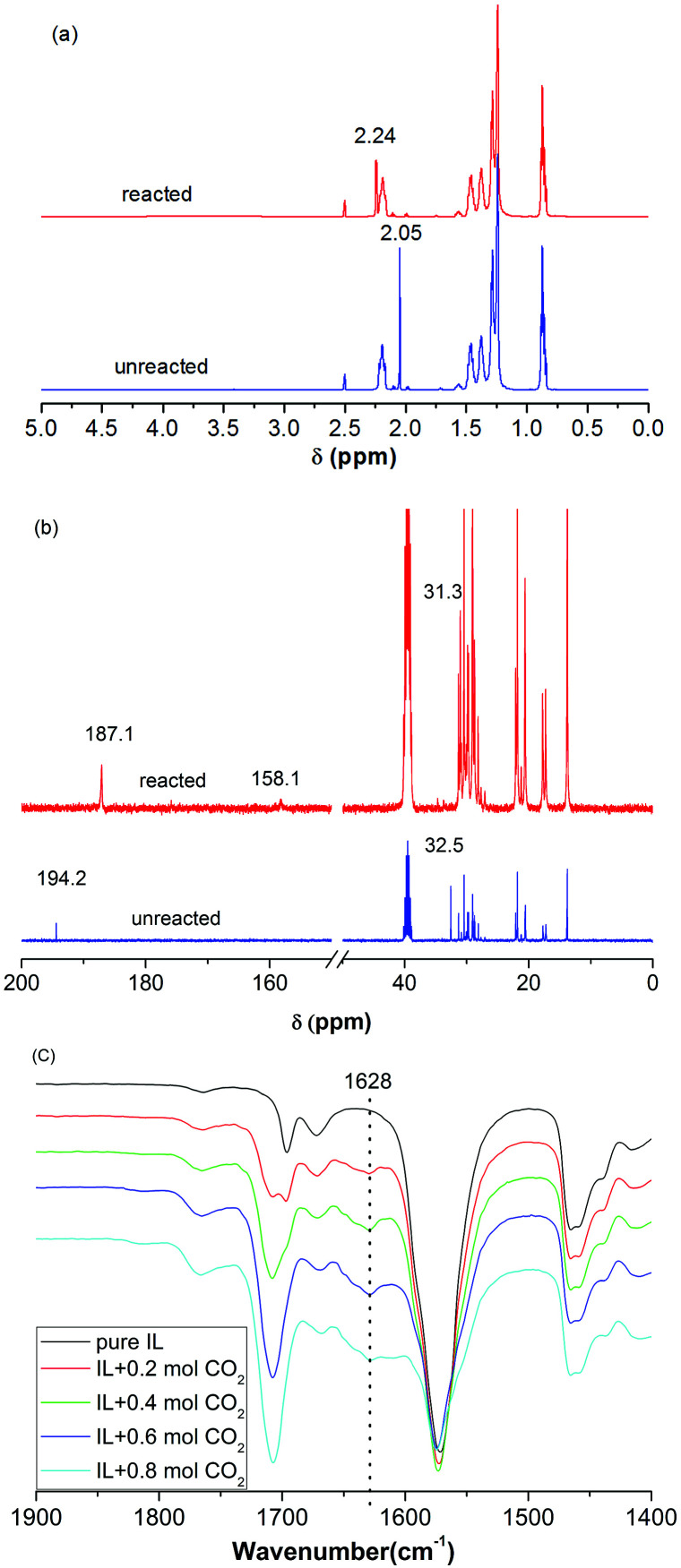
^1^H NMR (a), ^13^C NMR (b) and FT-IR (c) spectra of [P_66614_][H-Suc] before and after the absorption of CO_2_.

The interaction between CO_2_ and [P_66614_][H-Suc] was studied by ^1^H NMR, ^13^C NMR, and FT-IR spectra, and the results were illustrated in Fig. 4. Comparing the ^1^H NMR spectrum of CO_2_-IL with that of neat IL, it was found that the peak of 2× CH_2_ in [H-Suc]^−^ at *δ* = 2.05 ppm was shifted downfield to *δ* = 2.24 ppm after saturation of CO_2_ (Fig. 4a), indicating the strong interaction between CO_2_ and the anion of [P_66614_][H-Suc]. In the ^13^C NMR spectrum of CO_2_-IL, a new peak appeared at 158.1 ppm after CO_2_ uptake, this could be attributed to the formation of carbamate carbonyl carbon in N–CO_2_ interaction (Fig. 4b).^15,33^ On the other hand, when 0.2 to 0.8 mol CO_2_ was absorbed by [P_66614_][H-Suc], a new characteristic peak at 1628 cm^−1^ could be observed in the FT-IR spectrum of [P_66614_][H-Suc] (Fig. 4c), which belongs to the asymmetrical stretching vibration of N–CO_2_ due to the chemical interaction between CO_2_ and the electronegative N atom in [H-Suc]^−^ of the [P_66614_][H-Suc].^34^

The NMR and FT-IR spectra of [P_66614_][Ph-Suc] before and after the absorption of CO_2_ were showed in Fig. S1.† It can be seen that chemical shift of the protons in [Ph-Suc]^−^ from *δ* = 7.30–7.46 to 7.43–7.47 ppm was observed after CO_2_ absorption, while typical carbon peak of C=O in [Ph-Suc]^−^ moved from 185.0 ppm to 178.3 ppm, indicating the existence of chemical interaction between [Ph-Suc]^−^ and CO_2_. Moreover, the new characteristic peak at *δ* = 157.9 ppm after the absorption was attributed to the CO_2_ through the interaction of the N⋯CO_2_. No peak at about 1620 cm^−1^ could be seen from FT-IR spectrum after CO_2_ capture, which is possibly covered by other peaks. However, the peaks at 1619 and 1588 cm^−1^, which were assigned to the five-membered cyclic imide anion of [P_66614_][Ph-Suc], was blue shifted to 1769 and 1720 cm^−1^, respectively, suggesting the interaction between CO_2_ and N atom in the anion.^34^

**Scheme 1:**
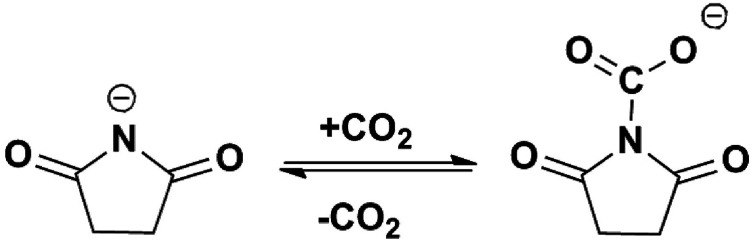
Possible mechanism of CO_2_ absorption by [P_66614_][H-Suc].

Based on the above solubility data and the spectroscopic investigation of the ILs before and after CO_2_ capture, the possible mechanisms of CO_2_ capture by [P_66614_][H-Suc] and [P_66614_][Ph-Suc] were proposed and shown in Schemes 1 and S1,† respectively, where the negatively charged N atom in the anions interacted with CO_2_ and formed the complexes.

### 3.3. The recycling of ILs for CO_2_ absorption

**Fig. 5:**
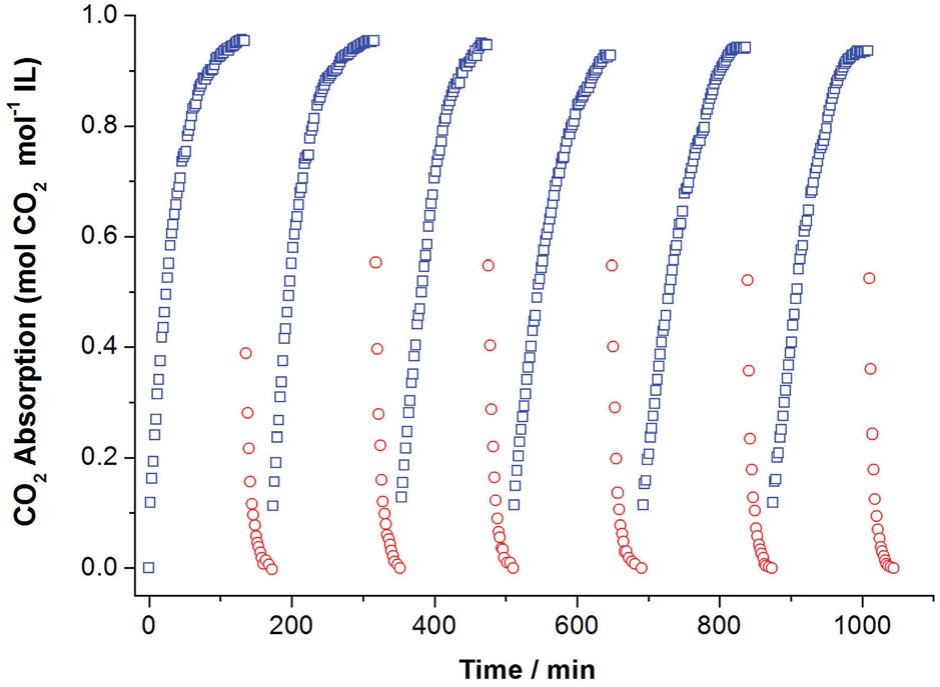
Six consecutive CO_2_ absorption–desorption cycles of [P_66614_][H-Suc]. The absorption of CO_2_ was performed at 308.15 K and 1.0 bar under CO_2_ (60 ml min^−1^). CO_2_ desorption was carried out at 333.15 K and 1.0 bar under N_2_ (60 ml min^−1^). Absorption, (□) desorption, (○).

Considering the fact that recycling performance of CO_2_ capture directly influences the application of ILs, we investigated CO_2_ absorption–desorption cycles by using [P_66614_][H-Suc] as an example (Fig. 5). It can be seen that the high CO_2_ absorption capacity and rapid absorption rate were remained during the 6 cycles, indicating that these amido-containing anion-functionalized ILs are highly recyclable.

### 3.4. Thermodynamic properties of CO_2_ absorbed in the ionic liquids

Thermodynamic properties such as the absorption Gibbs energy, enthalpy and entropy change are of great importance for the evaluation of new absorbents in practical application^35^ as well as for the understanding of thermodynamic driving force for the absorption.^27^ For example, absorption enthalpy can reflect the binding strength between the gas and the active site on the absorbent, absorption entropy can be used to probe the change in microstructure of the absorption systems. Thus, the thermodynamic properties of CO_2_ absorbed in these amido-containing anion-functionalized ILs were derived and analyzed. Usually, the absorption of CO_2_ in ILs consists of two parts, the physical absorption and the chemical absorption. Thus, the equations for physical CO_2_ capture and chemical CO_2_ capture can be expressed as follows:

1 CO_2_ (g) → CO_2_ (l)

2 CO_2_ (g) + IL (l) → CO_2_-IL (l)

where CO_2_ (g) and CO_2_ (l) in eqn (1) represent the CO_2_ in gaseous and liquid states, respectively, while IL (l) and IL-CO_2_ (l) in eqn (2) stand for the IL before and after CO_2_ capture. Since our functionalized ILs are basic and they can interact chemically with acid CO_2_ to form complex, IL-CO_2_ (l) in eqn (2) may represent such a complex.

Inspired by the previous reports,^25,26,28^ the CO_2_ absorption isotherm data in Fig. 2 and 3 were fitted with the “deactivated IL” model developed by Brennecke and co-workers.^25^ This model assumes that only 1 : 1 reaction takes place and less than 100% of the ionic liquids is allowed to react with CO_2_. The “deactivated IL” model can be expressed as follows:3
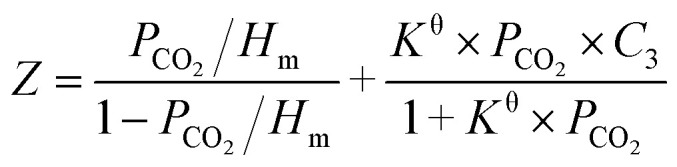
where *Z* is the solubility expressed by molar ratio of CO_2_ to ionic liquid. *K*^θ^, *P*_CO_2__, and *H*_m_ represent the equilibrium constant (dimensionless parameter), the CO_2_ partial pressure in kPa, and the Henry's constant in kPa, respectively. *C*_3_ stands for the molar ratio of active ionic liquids to the total ionic liquids. The first term denotes the contribution from physical absorption, while the second term is the contribution from chemical absorption.

It is known that when the pressure of CO_2_ is not higher than 100 kPa, the effect of CO_2_ physical absorption by IL on the absorption isotherms can be ignored.^25,26^ Considering the fact that our absorption isotherms of CO_2_ in the amido-containing anion-functionalized ILs were determined under the pressure up to 100 kPa and the CO_2_ absorption was mainly chemical, it is reasonable to ignore the CO_2_ physical absorption term in the fitting of absorption isotherms. Therefore, the CO_2_ absorption system by using these ILs as absorbents can be treated as an ideal chemical reaction system in the studied experimental temperature range, and the following equation of correlation between the solubility of CO_2_ in these ILs and partial pressure of CO_2_ could be obtained after simple derivation:4
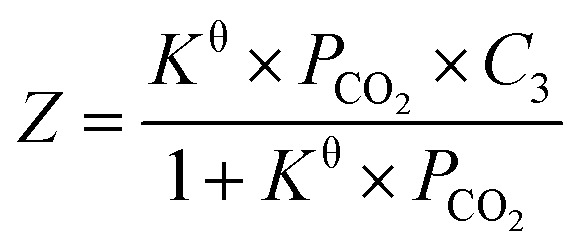


First, the values of the experimental solubility of CO_2_ in [P_66614_][H-Suc] and [P_66614_][Ph-Suc] were fitted to eqn (4) to obtain the values of chemical equilibrium constants at different temperatures, which were shown in the Table 1. It can be seen from Fig. 2 and 3 that the experimental solubility values of CO_2_ in the studied ILs could be well correlated with the CO_2_ partial pressure using eqn (4).

**Table 1:** Equilibrium constants for CO_2_ absorption in [P_66614_][H-Suc] and [P_66614_][Ph-Suc] at different temperatures

IL	Property	*T* (K)			
		308.15	313.15	318.15	323.15
[P_66614_][H-Suc]	*K* ^θ^	18.3 ± 1.4	9.7 ± 0.7	6.6 ± 0.8	3.2 ± 0.3
	*C*_3_	1.00 ± 0.02	0.87 ± 0.02	0.78 ± 0.03	0.62 ± 0.03
	*r* ^2^	0.992	0.994	0.984	0.990
[P_66614_][Ph-Suc]	*K* ^θ^	5.1 ± 0.3	4.5 ± 0.3	3.2 ± 0.2	2.4 ± 0.2
	*C*_3_	0.86 ± 0.02	0.64 ± 0.02	0.57 ± 0.02	0.50 ± 0.02
	*r* ^2^	0.998	0.996	0.997	0.996

**Fig. 6:**
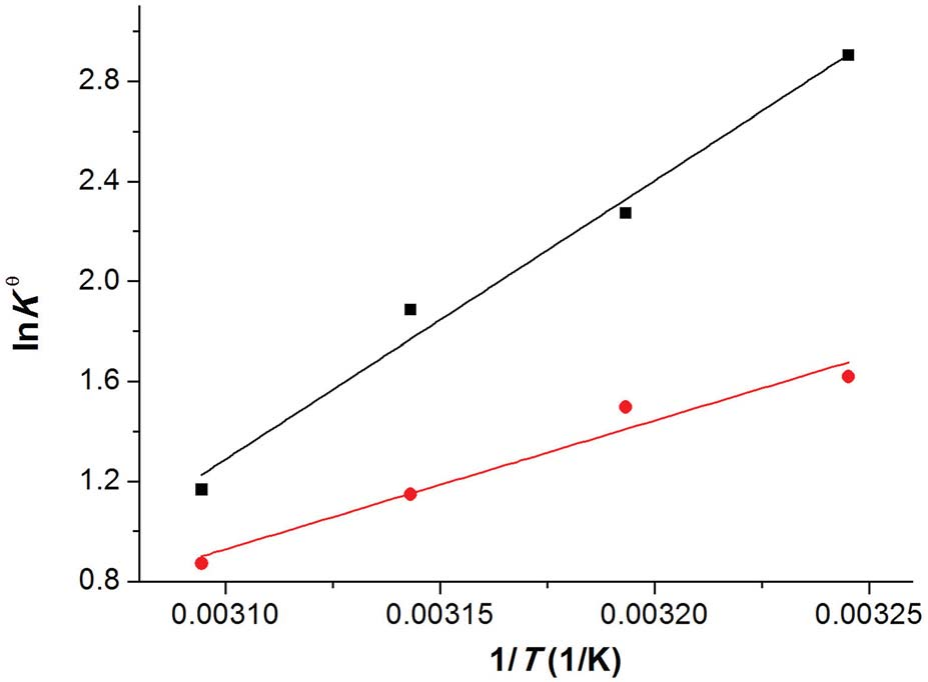
Linear correlation between ln *K*^θ^ and 1/*T*. [P_66614_][H-Suc], (■) [P_66614_][Ph-Suc], (●).

Since the temperature range studied in this work is quite narrow, the reaction enthalpy and reaction entropy for the CO_2_ absorption by the ILs could be determined by using the van't Hoff equation:5
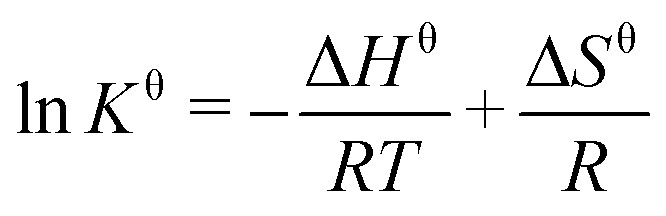
where Δ*H*^θ^ and Δ*S*^θ^ represent the enthalpy and entropy change for the chemical capture of CO_2_ in kJ mol^−1^ and kJ mol^−1^ K^−1^, respectively. *T* is the thermodynamic temperature in *K*, *R* denotes the universal gas constant (*R* = 8.314 J mol^−1^ K^−1^). The relationship between the logarithms of the equilibrium constant (*K*^θ^) and the reciprocal of temperature (1/*T*) was shown in Fig. 6. It can be seen that the linear relationship is reasonable for the CO_2_-[P_66614_][H-Suc] and CO_2_-[P_66614_][Ph-Suc] systems. From the slope and intercept of the straight lines, the Δ*H*^θ^ and Δ*S*^θ^ values could be obtained, and the results were given in Table 2.

**Table 2:** Molar reaction enthalpy, molar reaction Gibbs energy, and molar reaction entropy for the CO_2_ absorption by the amido-containing anion-functionalized ILs

IL	Property	*T* (K)			
		308.15	313.15	318.15	323.15
[P_66614_][H-Suc]	Δ*G*^θ^ (kJ mol^−1^)	−7.44	−5.92	−4.99	−3.13
	Δ*H*^θ^ (kJ mol^−1^)	−92.7			
	10^3^Δ*S*^θ^ (kJ mol^−1^ K^−1^)	−276.8			
[P_66614_][Ph-Suc]	Δ*G*^θ^ (kJ mol^−1^)	−4.15	−3.90	−3.03	−2.34
	Δ*H*^θ^ (kJ mol^−1^)	−42.9			
	10^3^Δ*S*^θ^ (kJ mol^−1^ K^−1^)	−125.2			

Then, the reaction Gibbs energy (Δ*G*^θ^) in kJ mol^−1^ could be calculated by the following equations:

6 Δ*G*^θ^ = −*RT* ln *K*^θ^

The resultant values of Δ*G*^θ^ were also collected in Table 2. It is clearly noted that Δ*H*^θ^ values for CO_2_-[P_66614_][H-Suc] and CO_2_-[P_66614_][Ph-Suc] systems are −92.7 kJ mol^−1^ and −42.9 kJ mol^−1^, respectively. The negative values indicate that the capture of CO_2_ is an exothermic process in [P_66614_][H-Suc] and [P_66614_][Ph-Suc] absorbents. Compared with the CO_2_-[P_66614_][Ph-Suc] system, Δ*H*^θ^ value of the CO_2_-[P_66614_][H-Suc] system is much lower, suggesting that the interaction between [P_66614_][H-Suc] and CO_2_ is much stronger than that between [P_66614_][Ph-Suc] and CO_2_. It is also implies that CO_2_ is more likely difficult to desorb from CO_2_-[P_66614_][H-Suc] than CO_2_-[P_66614_][Ph-Suc], and more energy consumption is needed in the regeneration of [P_66614_][H-Suc]. We also calculated the gas-phase reaction enthalpies of [H-Suc]-CO_2_ and [Ph-Suc]-CO_2_ complexes using Gaussian 09 program^36^ by DFT-D3(BJ) at the B3LYP/6-31++G(p,d) level. Although the calculated enthalpies were found to be only −38.4 and −24.3 kJ mol^−1^ for [H-Suc]-CO_2_ and [Ph-Suc]-CO_2_ complexes, respectively, the order was in agreement with the experimental result.

Furthermore, it can be seen from Table 2 that Δ*S*^θ^ has a negative value in the temperature range investigated, which indicates that the degree of disorder of the system becomes smaller due to the strong interaction of the IL with CO_2_ molecules and the formation of CO_2_-anion complexes mentioned above. The values of Δ*G*^θ^ are negative under the experimental conditions (Table 2), this is a strong indication that CO_2_ molecules are favourable to dissolve in the amido-containing anion-functionalized ILs. Moreover, considering the fact that Δ*G*^θ^, Δ*H*^θ^ and Δ*S*^θ^ are all negative, and absolute value of Δ*H*^θ^ is greater than *T*Δ*S*^θ^, the sign of Δ*G*^θ^ is determined by that of Δ*H*^θ^. Therefore, the enthalpy term is predominant for the favourable absorption of CO_2_.

## 4. Conclusion

Two kinds of amido-containing anion-functionalized ILs were synthesized, and evaluated for CO_2_ capture by CO_2_ solubility measurements at different temperatures and different CO_2_ partial pressures. It was found that high CO_2_ absorption capacity (up to 0.95 mol CO_2_ mol^−1^ IL) and low energy consumption regeneration of the ILs could be achieved by these anion-functionalized ILs. The interaction of negatively charged N atom in the anions with CO_2_ and the formation of CO_2_–anion complexes were responsible for the high absorption capacity. Thermodynamically, reaction enthalpy was the main driving force for CO_2_ capture. These results are useful for the design of new ionic liquid absorbents for capture of CO_2_ from flue gas.

## Conflicts of interest

There are no conflicts to declare.

## Supplementary Material

RA-009-C8RA07832G-s001
