# Supplementary material for: Absorption and thermodynamic properties of CO2 by amido-containing anion-functionalized ionic liquids
Source: RSC Adv. 2019 Jan 14;9(4):1882–8. doi: 10.1039/c8ra07832g (PMC9059720; doi:10.1039/c8ra07832g)
Supplement: RA-009-C8RA07832G-s001 [file RA-009-C8RA07832G-s001.pdf]

Electronic Supplementary Material (ESI) for RSC Advances.
This journal is © The Royal Society of Chemistry 2019

Electronic Supplementary Information

Absorption and thermodynamic properties of CO_2 in amido-containing anion-functionalized ionic liquids

Yanjie Huang, Guokai Cui, Huiyong Wang, Zhiyong Li, and Jianji Wang*

Henan Key Laboratory of Green Chemistry, Collaborative Innovation Center of Henan Province for Green Manufacturing of Fine Chemicals, Key Laboratory of Green Chemical Media and Reactions, Ministry of Education, School of Chemistry and Chemical Engineering, Henan Normal University, Xinxiang, Henan 453007, China.

E-mail: jwang@htu.cn

^{1}H NMR, ^{13}C NMR and FT-IR data of the amido-based ILs

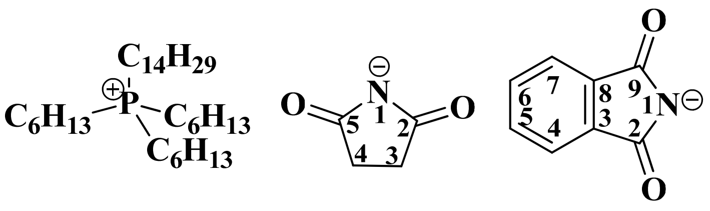

Numbering scheme for the positions in the amido-based anion-functionalized ILs

$[P_{66614}]$[H-Suc]:^{1}H NMR (DMSO-d_6): 0.86 (m, 12H, 4×CH_3), 1.236-1.469 (m, 48H, 24×CH_2), 2.05(s, 4H, C3 and C4), 2.19 (m, 8H, 4×PCH_2) ppm; ^{13}C NMR (DMSO-d_6): 13.8, 13.8, 17.3, 17.6, 20.5, 20.6, 21.2, 21.8, 21.9, 22.1, 28.1, 28.7, 28.7, 29.0, 29.0, 29.1, 29.7, 29.8, 30.0, 30.4, 30.8, 31.3, 32.5 (C3 and C4), 34.5, 35.5, 177.0, 174.4, 194.2 (C2 and C5) ppm; IR: 2955, 2923, 2854, 1764, 1696, 1672, 1572, 1465, 1416, 1377, 1315, 1273, 1240, 1211, 1163, 1112, 1003, 987, 891, 857, 821, 720 cm^{-1}.

$[P_{66614}]$[Ph-Suc]:^{1}H NMR(DMSO-d_6): 0.86 (m, 12H, 4×CH_3), 1.237-1.464 (m, 48H, 24×CH_2), 2.18 (m, 8H, 4×PCH_2), 7.30 - 7.36 (m, 4H, C4, C5, C6, and C7) ppm; ^{13}C NMR (DMSO-d_6): 13.7, 13.8, 17.3, 17.6, 20.5, 20.6, 21.2, 21.8, 21.9, 22.1, 28.1, 28.7, 28.7, 29.0, 29.0, 29.1, 29.7, 29.8, 30.0, 30.4, 30.8, 31.3, 119.0 (C4 and C7), 125.8, 128.6, 129.3 (C5 and C6), 129.5, 129.9, 130.9, 139.6 (C3 and C8), 145.0, 169.4, 172.9, 185.0 (C2 and C9) ppm; IR: 2955, 2924, 2854, 1698, 1662, 1619, 1588, 1571, 1465, 1460, 1409, 1378, 1364, 1343, 1273, 1202, 1178, 1158, 1125, 1004, 861, 831, 814, 721 cm^{-1}.

Table S1. Solubility (n_{CO2}) of CO_2 in [P_{66614}][H-Suc] at different temperatures (T) and CO_2 partial pressures (P_{CO2}).

P_{CO2} (100 kPa)	n_{CO2} (mol CO_2 mol^{-1} IL)	P_{CO2} (100 kPa)	n_{CO2} (mol CO_2 mol^{-1} IL)
T = 308.15 K		T = 313.15 K	
0.025	0.36	0.025	0.16
0.05	0.50	0.05	0.26
0.075	0.57	0.075	0.35
0.10	0.61	0.10	0.43
0.15	0.70	0.15	0.52
0.20	0.76	0.20	0.57
0.25	0.82	0.25	0.64
0.30	0.86	0.30	0.68
0.50	0.92	0.50	0.73
0.70	0.94	0.70	0.74
1.00	0.95	1.00	0.76
T = 318.15 K		T = 323.15 K	
0.025	0.10	0.025	0.04
0.05	0.16	0.05	0.07
0.075	0.21	0.075	0.11
0.10	0.32	0.10	0.14
0.15	0.40	0.15	0.19
0.20	0.45	0.20	0.24
0.25	0.50	0.25	0.29
0.30	0.57	0.30	0.32
0.50	0.60	0.50	0.41
0.70	0.61	0.70	0.43
1.00	0.66	1.00	0.45

Table S2. Solubility (n_{CO2}) of CO_2 in [P_{66614}][Ph-Suc] at different temperatures (T) and CO_2 partial pressures (P_{CO2}).

P_{CO2} (100 kPa)	n_{CO2} (mol CO_2 mol^{-1} IL)	P_{CO2} (100 kPa)	n_{CO2} (mol CO_2 mol^{-1} IL)
T = 308.15 K		T = 313.15 K	
0.05	0.18	0.05	0.10
0.10	0.30	0.10	0.19
0.15	0.37	0.15	0.26
0.25	0.46	0.25	0.34
0.30	0.52	0.30	0.38
0.50	0.61	0.50	0.45
0.70	0.66	0.70	0.49
1.00	0.73	1.00	0.52
T = 318.15 K		T = 323.15 K	
0.05	0.06	0.05	0.04
0.10	0.13	0.10	0.09
0.15	0.19	0.15	0.13
0.25	0.25	0.25	0.19
0.30	0.28	0.30	0.22
0.50	0.35	0.50	0.27
0.70	0.41	0.70	0.31
1.00	0.47	1.00	0.38

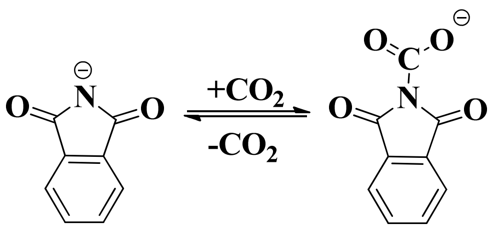

Scheme S1. Possible mechanism of CO_2 absorption by [P_{66614}][Ph-Suc].

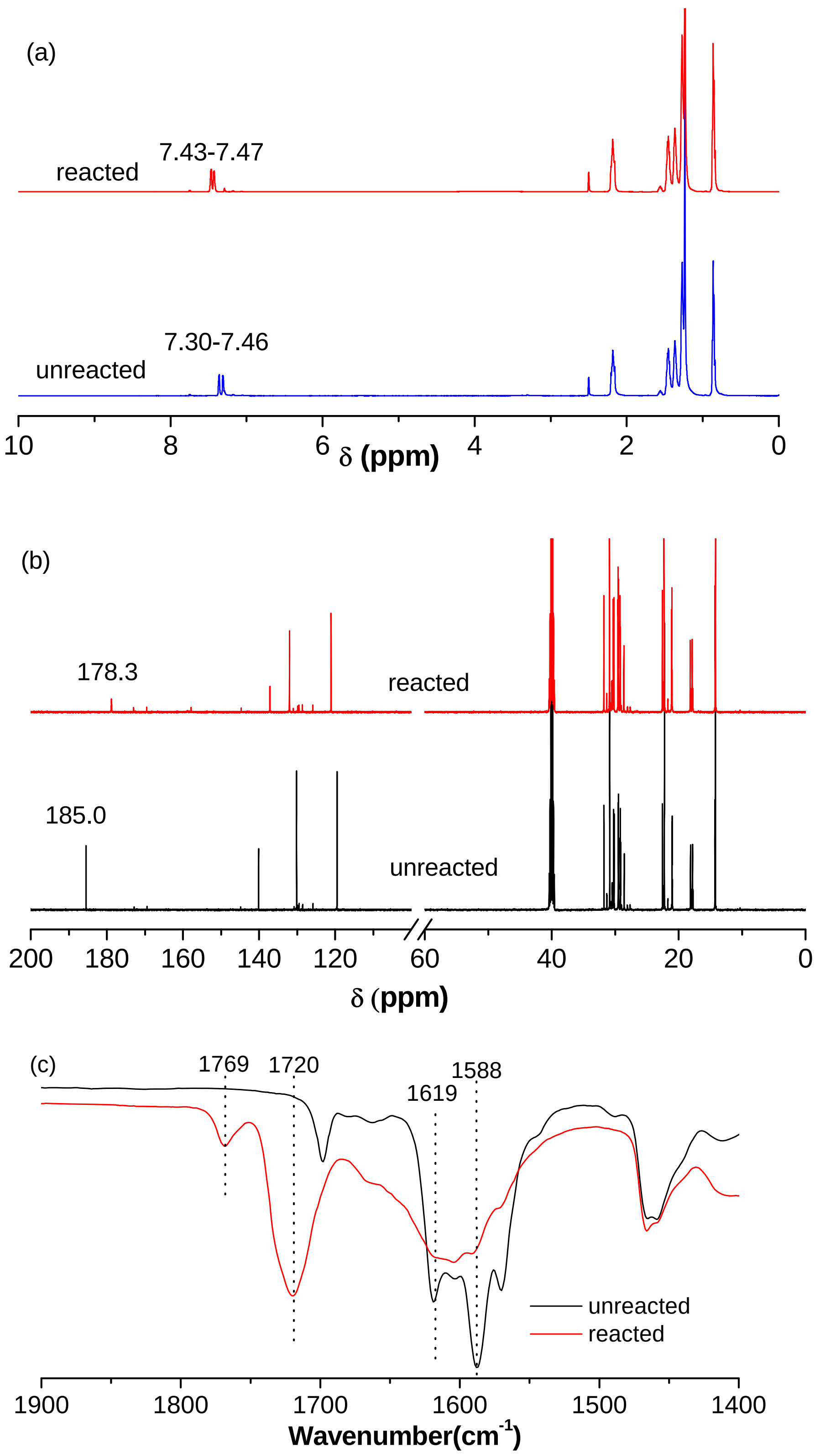

Figure S1. ^{1}H NMR (a), ^{13}C NMR (b) and FT-IR (c) spectra of $[P_{66614}]$[Ph-Suc] before and after absorption of CO_2.
